# A Novel Mouse Model of Ischemic Carotid Artery Disease

**DOI:** 10.1371/journal.pone.0100257

**Published:** 2014-06-18

**Authors:** Yorito Hattori, Akihiro Kitamura, Kazuyuki Nagatsuka, Masafumi Ihara

**Affiliations:** 1 Department of Regenerative Medicine and Tissue Engineering, National Cerebral and Cardiovascular Center, Suita, Osaka, Japan; 2 Department of Neurology, Kyoto University Graduate School of Medicine, Kyoto, Japan; 3 Department of Stroke and Cerebrovascular Diseases, National Cerebral and Cardiovascular Center, Suita, Osaka, Japan; Massachusetts General Hospital/Harvard Medical School, United States of America

## Abstract

**Background:**

Carotid artery occlusive disease gradually develops over time, eventually leading to cerebral infarction and high mortality rate. Animal models replicating cerebral infarction resulting from carotid artery occlusive disease have thus been developed to test potential novel treatments, which could be subsequently administered clinically.

**Methods:**

Adult C57BL/6J male mice were subjected to ameroid constrictor (AC) placement to gradually narrow the bilateral common carotid arteries. Cerebral blood flow (CBF) was measured at several time points. At 7 and 28 days post-operation, post-mortem brain samples were analyzed for ischemic changes.

**Results:**

The mortality rate was 58.8% at 28 days post-operation. Surviving mice with AC showed continuous reduction of CBF by up to 70% of the baseline level at 28 days. Most of the mice (75%) showed multiple cerebral infarctions in the gray and white matter. Non-surviving mice showed critical CBF reduction below 20–30% of the baseline level before death.

**Conclusion:**

The application of the AC on the bilateral common carotid arteries in mice could offer a reliable model of severe cerebrovascular insufficiency due to carotid artery occlusive disease and may thus be useful in exploring pharmacological intervention in stroke through monitoring survival rate, infarct formation, and CBF profile.

## Introduction

Number of patients with severe carotid artery diseases is gradually increasing because metabolic disorders are prevalent worldwide with the adoption of a sedentary lifestyle combined with excessive caloric intake. They are susceptible to cerebral ischemia such as cerebral infarction [1] and cognitive impairment [2]. Patients with bilateral carotid artery occlusion have a particularly poor prognosis as a result of a high subsequent stroke prevalence rate (66% in bilateral internal carotid artery occlusion and 71% in bilateral common carotid artery occlusion [3]), and high mortality rate of more than 50% over six years [4]. Therefore, animal models which can mimic natural history of severe carotid artery disease are warranted to develop novel and safer prophylactic medications to protect patients at risk from cerebral ischemia distal to severe carotid artery diseases.

Several animal models of cerebral infarctions have been established including models of transient/permanent middle cerebral arterial occlusion and embolic stroke [5], with an infarction-inducing model of severe carotid artery disease developed in Mongolian gerbils [6]. Nevertheless, the suitability of the gerbil model of cerebral infarction distal to bilateral common carotid artery stenosis has been questioned due to an incomplete circle of Willis, absence of the posterior communicating artery (PcomA) [7] in such species. Furthermore, as gerbils are seizure-prone animals, generalized tonic-clonic seizures tend to occur spontaneously or in response to environmental stimuli including cerebral ischemia and may alter cerebral blood flow after seizures [8]. Besides, all C57BL/6J and almost all of Institute of Cancer Research (ICR) and BALB/c mice subjected to ligation of bilateral common carotid arteries (CCAs) die within 24 hours [9]. Thus, a suitable mouse model of cerebral infarction by carotid artery stenosis/occlusion has not been established.

We thus propose a mouse model that successfully induces severe cerebrovascular insufficiency and multiple cerebral infarctions using ameroid constrictors (AC) placed on the bilateral CCA.

## Materials and Methods

### Ethical Statement

All procedures in this study were carried out in strict accordance with the guidelines for animal experimentation from the Animal Research Committee of Kyoto University and that of National Cerebral and Cardiovascular Center. The protocol was approved by the Animal Research Committee, Kyoto University (Permit Number: MedKyo13270), and National Cerebral and Cardiovascular Center (Permit Number: 13055). All surgery was performed under anesthesia, and all efforts were made to minimize suffering.

### Ameroid Constrictor (AC)

The AC consists of a titanium casing surrounding a hygroscopic casein material with an internal lumen (Tokyo Instruments). The casein component gradually absorbs water and consequently swells, leading to predictable narrowing and occlusion of the arterial lumen it encases. The inner diameter was 0.5 mm, the outer diameter 3.25 mm, and the length 1.28 mm [10].

### Experimental Protocol

Male C57BL/6J mice of 10–12 weeks of age (CLEA Japan) were assigned into three groups: (1) sham-operation group (n = 6), (2) AC group (n = 29) [10] and (3) bilateral common carotid artery stenosis (BCAS) group (n = 7) [11]. Cerebral blood flow (CBF) was monitored before and at 2 hours, 1 day, 3 days, 7 days, 14 days and 28 days after operation. After 7 and 28 days following each operation, the mice were humanely euthanized by transcardial perfusion fixation with 4% paraformaldehyde after they were completely static and unresponsive to a toe pinch under anesthesia with intraperitoneal injection of 40 mg/kg pentobarbital. The brains were removed and post-mortem brains were analyzed for ischemic changes with hematoxylin and eosin (H&E) staining and immunohistochemistry for a rabbit antiglial fibrillary acidic protein antibody (GFAP, a marker of astrocyte) and a rabbit anti-Iba1 antibody (a marker of microglia). All mice were housed in a room with a 12-hour light/dark cycle (lights on at 7∶00 a.m.) and were given access to food and water *ad libitum*. We have monitored the condition of the animals every day until 14th day after operation, and twice per week after 14th day.

### Surgical Procedure

Under anesthesia with 1.5% isoflurane, operation was conducted after confirming the mice being completely static and unresponsive to a toe pinch. Both CCAs were exposed through midline cervical incision and freed from their sheaths. In the AC group, the ACs were applied to the bilateral CCAs; while in the BCAS group, mice were subjected to surgical implantation of microcoils with an inner diameter of 0.18 mm to the bilateral CCAs [11]. Rectal temperature was maintained between 36.5°C and 37.5°C by a self-regulating heating pad.

### Measured CBF by Laser Speckle Flowmetry

Relative CBF was recorded by laser speckle flowmetry (Omegazone, Omegawave), which produces high-resolution, two-dimensional imaging with a linear relationship to absolute CBF values [12]. Recordings were performed under anesthesia with 1.5% isoflurane. The scalp was removed by a midline incision so that the skull was exposed throughout the experiment. During the measurement of CBF, the skull surface was illuminated by 780 nm of laser light. The scattered light was filtered and detected by a CCD camera positioned over the head. The filter detected only scattered light that had a perpendicular polarization to the incident laser light. The raw speckle images were used to compute speckle contrast, which corresponds to the measured velocity of moving red blood cells thus approximating CBF. Signal processing was performed by an algorithm developed by Forrester et al. [13]. Color-coded blood flow images were obtained in high-resolution mode (639×480 pixels; 1 image/sec) and the sample frequency was 60 Hz. One blood flow image was generated by averaging numbers obtained from 20 consecutive raw speckle images. The recordings were initiated after the examiner confirmed that CBF did not change over 1 min, and the five recordings of blood flow image were averaged. In order to prevent the fluctuation of CBF and blood pressure during the measurement of CBF, anesthesia was induced, as described above. During the measurement of CBF, mice were held in a small plastic holder on a warming pad and thermostatically controlled at 36.5°C to 37.5°C in rectal temperature. Blood pressure was measured by the tail cuff method (Softron), which confirmed consistency throughout the experiment.

### Histologic Evaluation

Paraffin-embedded coronal sections of the brain (6-µm-thick) were analyzed with H&E staining and immunohistochemistry for GFAP (1∶2000; DAKO) and Iba1 (1∶200; Wako) for detection of infarct areas at the level of the forebrain (bregma) and the hippocampus (+2 mm from bregma). The number of infarcts was counted in the cerebral cortex, the corpus callosum, the caudoputamen, the anterior commissure, the hippocampal fimbria, and the hippocampus. Infarcts were defined as focal areas of rarefaction that were accompanied by a group of astrocytes or microglia/macrophage proliferation.

### Humane Endpoints during the Survival Study

We used moribund conditions as humane endpoints during the survival study. The moribund condition was defined as irreversible conditions leading inevitably to death. Signs of moribundity included a) lack of responsiveness to manual stimulation; b) immobility; and/or c) an inability to eat or drink. In such conditions, animals were euthanized by carbon dioxide asphyxiation.

### Statistical Analysis

All values are expressed as means±standard error of the mean (SEM) in the text and figures. Differences with *p<*0.05 were considered statistically significant in all statistical analyses used, Student’s t-test and two-way repeated-measures analysis of variance (ANOVA).

## Results

### Temporal Profiles of CBF Recorded by Laser Speckle Flowmetry

**Figure 1:**
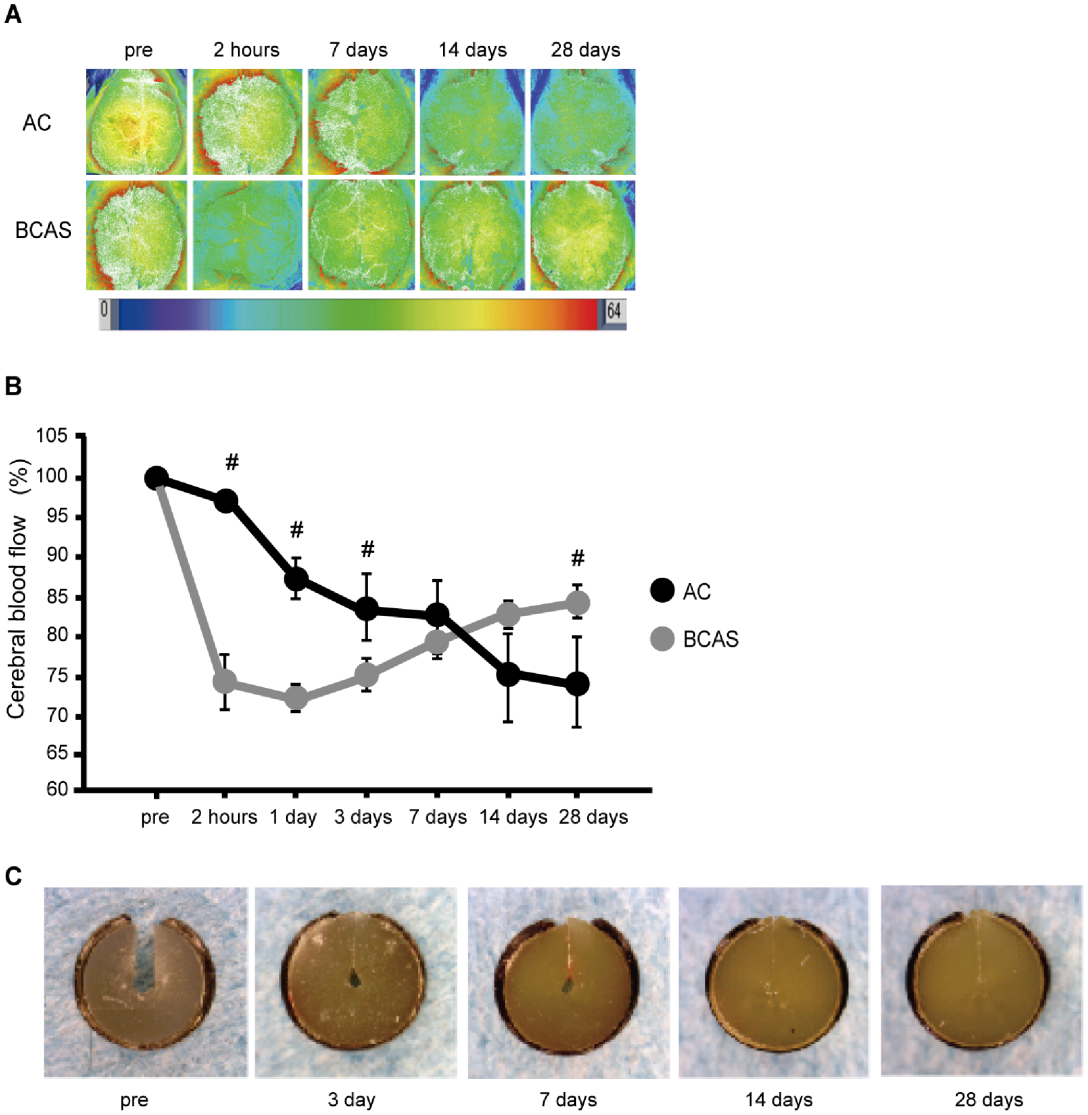
Cerebral blood flow (CBF) was gradually decreased in mice with the ameroid constrictors (ACs). (A) Representative CBF images of AC-implanted mice and bilateral common carotid artery stenosis (BCAS)-operated mice as assessed by laser speckle flowmetry at indicated time points. (B) Temporal profiles of CBF of the AC-implanted mice (n = 8) and BCAS-operated mice (n = 7) pre- and post-operation (2-way repeated-measures ANOVA, *p*<0.05; unpaired t-test, #*p*<0.01 vs. BCAS group). (C) Representative images of ACs at indicated time points.

At 2 h, 1 day, and 3 days post-operation, the percent of the baseline CBF value of the AC group was significantly preserved compared with that of BCAS group (AC vs. BCAS; 2 h, 98.2±0.1% vs. 74.9±4.3%; 1 day, 87.5±2.2% vs. 73.1±1.3%; 3 days, 85.1% ±3.9% vs. 75.2±1.5%). However, the CBF continued to decrease at least up to 28 days after the operation in the AC group while CBF started to recover at 3 days after the operation in the BCAS group. Eventually, the CBF of the AC-implanted mice significantly decreased compared with that of BCAS-operated mice at 28 days post-operation (AC, 74.7±5.4% vs. BCAS, 85.1±1.4%) (Fig. 1A and B). In the sham-operation group, no apparent change of CBF was detected by 28 days post-operation (data not shown). Bilateral placement of the ACs on the CCAs produced the expected narrowing of the inner lumen and finally the inner lumen was occluded without any corrosion or infection of ACs at 28 days after operation (Fig. 1C).

### Histologic Changes after Surgical Implantation of ACs

**Figure 2:**
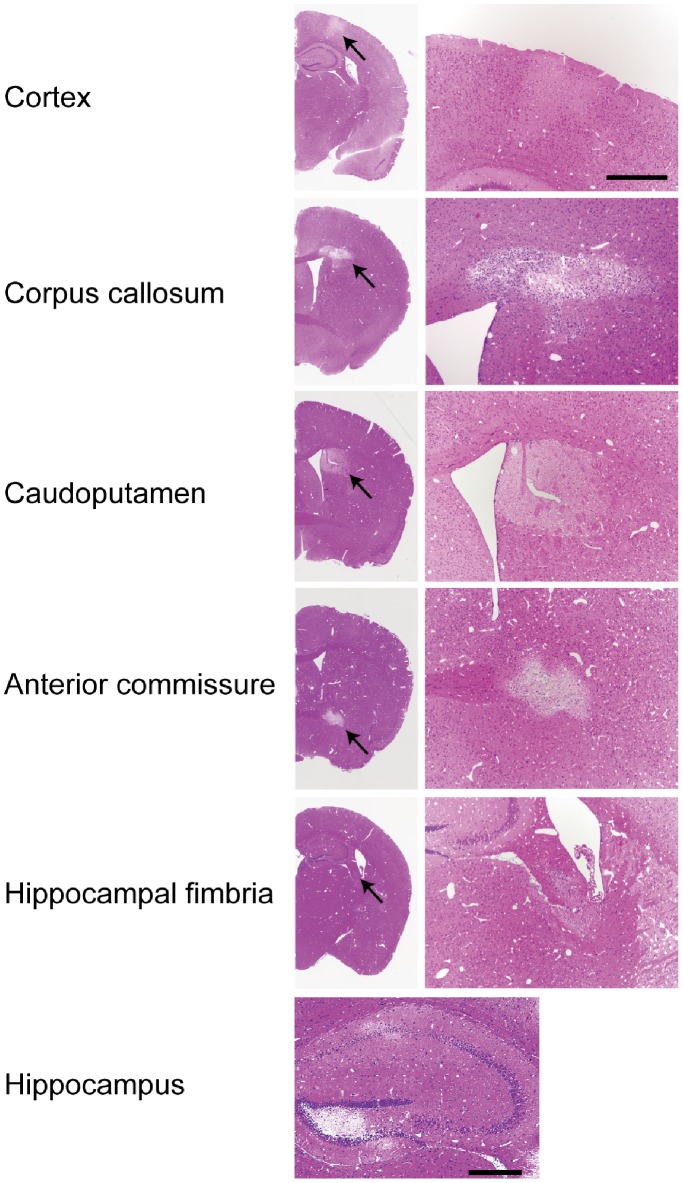
Multiple cerebral infarcts in mice with ACs. Hematoxylin and eosin staining showed multiple infarcts in the cerebral cortex, the corpus callosum, the caudoputamen, the anterior commissure, the hippocampal fimbria, and the hippocampus of the mice in the AC group at 28-operation. Arrows indicate infarct changes. Scale bars indicate 200 µm.

**Figure 3:**
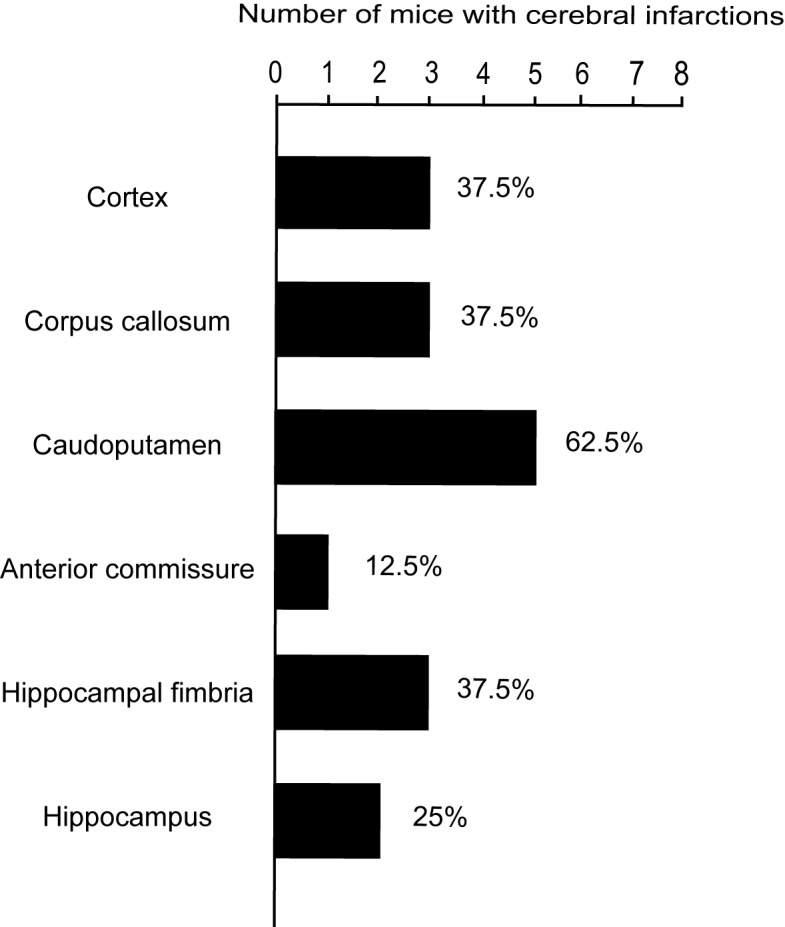
Percentage of AC-implanted mice with cerebral infarctions. The histogram shows the number and percentage of AC-implanted mice (n = 8) that developed cerebral infarctions in cortex, corpus callosum, caudoputamen, anterior commissure, hippocampal fimbria, and hippocampus.

BCAS-operated mice did not show any cerebral infarction at 7 and 28 days after operation as previously reported [11]. In AC-implanted mice, cerebral infarctions did not develop at 7 days after operation, but developed in 6 of 8 mice (75%) at 28 days after operation. Infarctions occurred in the cerebral cortex, the corpus callosum, the caudoputamen, the anterior commissure, the hippocampal fimbria, and the hippocampus (Table 1 and Fig. 2, 3). The size of infarcts in the cortex tended to be smaller, which ranged from 100–200 µm in diameter while those in the other areas ranged from 300–800 µm with greater rarefaction. Some of the infarcts in the cerebral cortex were found in the arterial borderzone between anterior and middle cerebral arteries.

**Table 1:** Localization of cerebral infarctions of the ameroid constrictor-implanted mice.

mouse	Bregma Level				Hippocampal level				
No.	Cx	CC	CPu	ACo	Cx	CC	CPu	F	H
1	1		1		1				
2									
3	1	1	3		1			2	3
4	1				1				
5			1						
6		1	1	1		1	1	1	
7		1				1	1	2	1
8									

Cx, cortex; CC, corpus callosum; CPu, caudoputamen; ACo, anterior commissure; F, Hippocampal fimbria; H, hippocampus.

**Figure 4:**
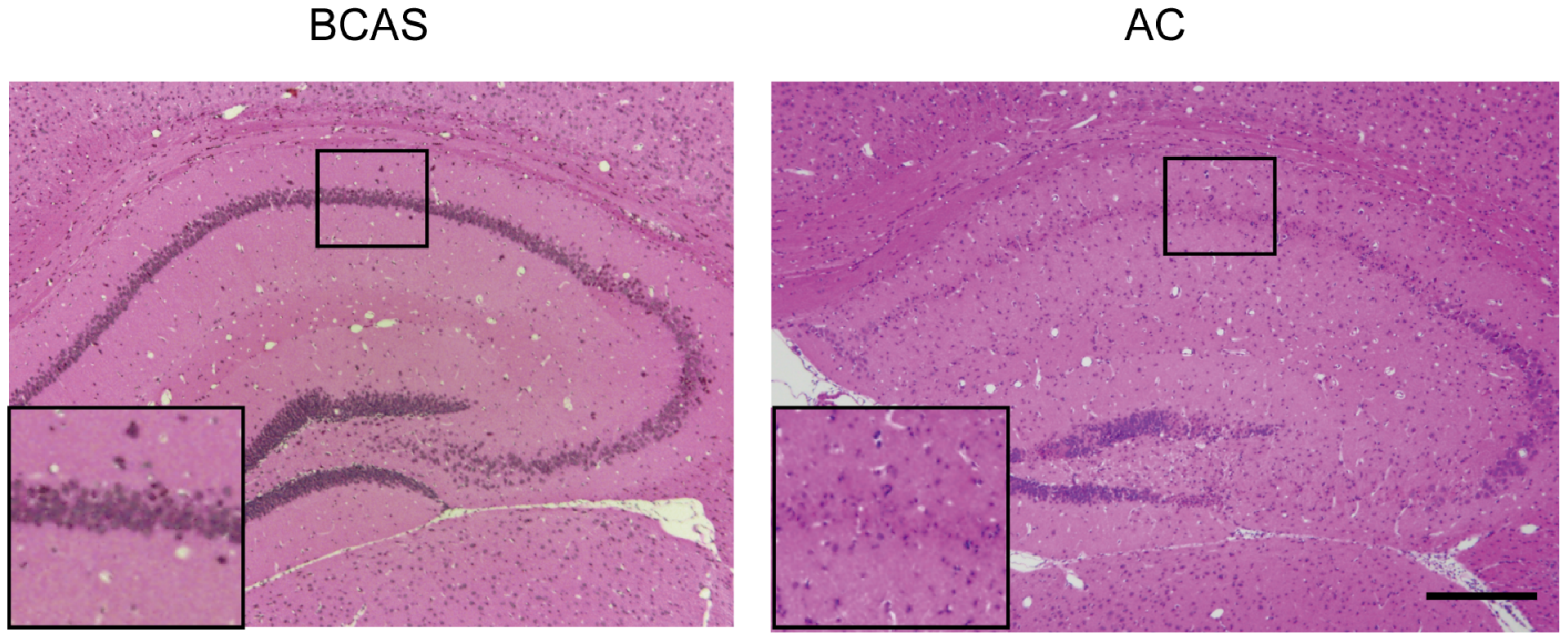
Hippocampal neuronal loss in AC-implanted mice. Hematoxylin and eosin staining showed intact hippocampus of the BCAS-operated mouse (left panel) and hippocampal neuronal loss of the AC-implanted mouse (right panel) at 28 days post-operation. The inset shows a magnified image of the indicated area. Scale bar indicates 200 µm.

Furthermore, in 5 of 8 mice, hippocampal neuronal loss was observed (Fig. 4). By contrast, BCAS-operated mice did not show any hippocampal damage as previously reported [11].

### Mortality Rate

**Figure 5:**
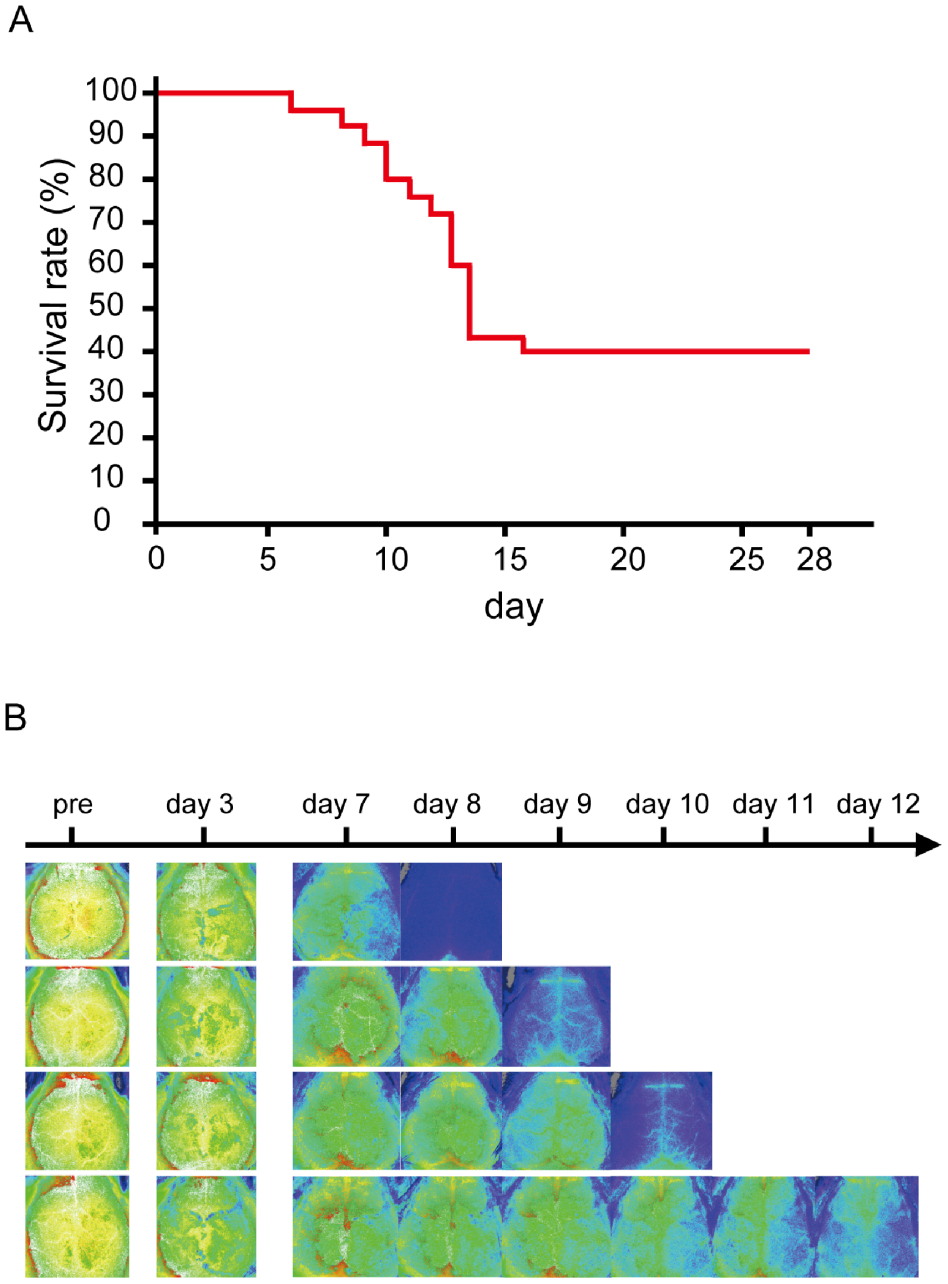
Survival rate in the AC group. (A) Kaplan-Meier method indicates survival rate of mice in the AC group (n = 29). (B) CBF images of the 4 non-surviving mice in the AC group which died during the second week. These mice died on the next day of last CBF measurement.

The mortality rate at 28 days after implantation of ACs was 58.8% (Fig. 5A) while all BCAS-operated mice survived. Most of the non-surviving mice with ACs died during the second week; the LSF showed significant reductions of CBF below 20–30% of the baseline level reflecting massive infarctions or hemodynamic derangements (Fig. 5B).

## Discussion

This study proposes a novel mouse model of carotid artery occlusive disease which showed that (1) ACs gradually narrowed and finally occluded the bilateral CCAs by 28 days post-operation; (2) CBF gradually decreased up to 28 days post-operation without acute CBF drop; (3) multiple cerebral infarctions were induced in the gray and white matter of most animals; and (4) the mortality rate was 58.8%, which may reflect severe pathophysiology of carotid artery occlusive disease like in humans in which the mortality rate is high [4].

The BCAS model is currently thought to be the most promising animal model of chronic cerebral hypoperfusion [14], [15], but one of the limitations is an acute drop of CBF by approximately 30% and gradual CBF recovery due to compensatory mechanism [11]. To circumvent this limitation, models were developed by placing ACs to the bilateral CCAs of Wister-Kyoto rats, which reproduced gradual CBF reduction, chronic cerebral hypoperfusion and specific white matter changes [10]. However, because of the functioning PcomA, Wistar-Kyoto rats do not develop any cerebral infarcts even after complete occlusion of bilateral CCAs.

In the current C57BL/6J mouse model, however, we could reproduce several characteristics of carotid artery occlusive disease of humans. First, gradual narrowing of CCAs partly simulates consequences of atheromatous plaques that gradually enlarge in the carotid arteries. Second, ischemic infarcts were induced after the AC implantation as often observed in human carotid artery occlusive disease [3], [4]. Compared to the rats or other mouse strains, the C57BL/6 mice have less developed PcomA and are thought to be the most susceptible to cerebral ischemia following bilateral common carotid occlusion/stenosis [7]. Such background characteristics may have contributed to the successful modeling of carotid occlusive disease with infarcts in mice. Therefore, the current model differs from the BCAS model in terms of severity of histological changes because BCAS model induces white matter rarefaction but not infarcts while the current AC model is characterized by ischemic infarcts in the gray and white matter as a result of greater CBF reductions at later phase post-operation. Therefore, this model will be used to elucidate the mechanism of CBF autoregulation observed in humans and how this mechanism fails when systemic arterial pressure decreases below a critical point [16]. This would also be useful to investigate the dynamics of interstitial fluid and cerebrospinal fluid of the brain [17] as well as to test new therapeutic strategies for the large spectrum of neurological conditions associated with cerebral ischemia.

This study has a limitation in that the detailed mechanism of infarct formation remains unknown. In humans, cerebral infarctions distal to severe carotid artery diseases are caused by hemodynamic impairment and/or artery-to-artery embolism [1]. In the current mouse model without atheromatous lesions, although hemodynamic mechanism may be more plausible, detailed mechanisms remains unclear. Further analysis of the underlying mechanism of infarct formation in carotid artery occlusive disease can be conducted by using this novel animal model.

Another limitation is the relatively high mortality rate of the current model raising an issue of survival bias when future intervention study will be conducted with histological or behavioral assessment. However, since severe hemodynamic derangement itself seems to be a direct cause of mortality, death may become an endpoint and survival rate will be compared between the experimental groups to overcome the inherent limitation of the survival bias.

In summary, we have successfully established a mouse model of severe cerebrovascular insufficiency which almost invariably induce cerebral infarctions by placing ACs on the bilateral CCAs. We anticipate that pharmacological intervention for stroke will be investigated using this model by evaluation of survival rate, infarct formation, and CBF profile after the operation.
